# Draft genome sequencing of *Enterococcus faecium* MBBL3, a probiotic strain isolated from healthy cow milk

**DOI:** 10.1128/mra.00926-24

**Published:** 2024-12-10

**Authors:** Naim Siddique, Md. Morshedur Rahman, Monira Rahaman, A. N. M. Aminoor Rahman, Anup Kumar Talukder, Ziban Chandra Das, M. Nazmul Hoque

**Affiliations:** 1 Molecular Biology and Bioinformatics Laboratory, Department of Gynecology, Obstetrics and Reproductive Health, Bangabandhu Sheikh Mujibur Rahman Agricultural University (BSMRAU), Gazipur, Bangladesh; University of Maryland School of Medicine, Baltimore, Maryland, USA

**Keywords:** draft genome, *Enterococcus faecium*, probiotics, cow milk

## Abstract

We sequenced the whole genome of *Enterococcus faecium* MBBL3, isolated from healthy cow milk. The draft genome of MBBL3 is 2,971,195 bp with 95.61× coverage, spanned over 106 contigs.

## ANNOUNCEMENT

*Enterococcus*, the third largest genus of lactic acid bacteria, is recognized for its production of antimicrobial proteins and peptides known as bacteriocins, a trait increasingly valued in probiotics (1, 2). *Enterococcus faecium*, a prominent species within the genus *Enterococcus*, has been extensively studied and recognized for its probiotic potential. It is utilized as a probiotic in both livestock and human health applications (3, 4), owing to its beneficial effects on host microbiota and overall health (5, 6).

We isolated *E. faecium* MBBL3 from a milk sample obtained from a healthy cow at the BSMRAU dairy farm (24.09° N, 90.41° E) in Gazipur, Bangladesh. During morning milking (8:00–10:00 a.m.), approximately 10 mL of milk was aseptically collected from a cow in a sterile Falcon tube, with meticulous attention to pre-sampling disinfection of teat ends and rigorous adherence to hygiene protocols. To isolate and phenotypically identify *E. faecium*, 1 mL of milk sample was initially enriched in 9 mL of nutrient broth (Oxoid, UK) and incubated overnight at 37°C. The bacteria-enriched broth was then serially diluted up to 10^−3^-fold and plated on nutrient agar (Oxoid, UK), followed by overnight incubation at 37°C. Pure colonies were obtained by streaking on selective MRS (De Man, Rogosa, and Sharpe) agar (Oxoid, UK) plates by incubating at 37°C for 36 to 48 hrs (7, 8). Phenotypic identification was carried out based on colony morphology, Gram staining, and biochemical tests (e.g., indole, Voges-Proskauer, oxidase, urease, and citrate tests). This isolate was confirmed as *E. faecium* through a VITEK-2 system v9.01 (9). The MBBL3 isolate was subcultured in nutrient broth (Biolife, Italy) overnight at 37°C, followed by DNA extraction using QIAamp DNA Mini kit (QIAGEN, Germany). The purity and concentration of the extracted DNA were assessed using a NanoDrop 2000 UV-Vis Spectrophotometer (Thermo Fisher, USA). We used the Nextera DNA Flex library preparation kit (Illumina, San Diego, USA) to generate libraries from 1 ng of DNA and performed whole-genome sequencing on the prepared libraries using the Illumina MiSeq sequencer (Illumina, USA) with a 2 × 250 bp protocol (10). The raw reads of MBBL3 (5,046,808 bp) underwent trimming using Trimmomatic v0.39 (11) to remove Illumina adapters, known Illumina artifacts, and phiX reads, followed by quality assessment with FastQC v0.12.1 (12). Assembly of reads with Phred scores greater than 20 was performed using SPAdes v.3.15.5 (13), and the quality of the assembled genome was evaluated using QUAST v.5.2.0 (14). Genome annotation was performed using the Prokaryotic Genome Annotation Pipeline v.6.6 (15), and the completeness of the gene set was assessed with the *E. faecium* CheckM marker set (16). To predict antimicrobial resistance genes (ARGs), virulence factor genes (VFGs), plasmids, prophages, and metabolic functions in the draft genome, we employed ResFinder 4.0 (17), VFDB v6.0 (18), PlasmidFinder (19), PHASTER (20), and RAST (21) databases, respectively. Default parameters were used in all analyses unless otherwise specified.

The draft genome assembly of MBBL3 consists of 2,971,195 bp, with comprehensive sequencing and assembly metrics provided in Table 1. The genome analysis revealed two ARGs, *aac(6')-II* and *msr(C*), along with two plasmids. However, no VFGs were identified in MBBL3. Additionally, seven prophage regions were predicted using the PHASTER tool. The genome exhibited 227 metabolic features distributed across various subsystem categories. Furthermore, a bacteriocin gene cluster encoding Enterolysin_A was detected using the BAGEL4 tool (22), alongside four secondary metabolite biosynthetic gene clusters identified through AntiSMASH 7.0 (23). Therefore, *E. faecium* MBBL3, isolated from healthy cow milk and lacking specific ARGs and VFGs, holds a potential as a bioactive probiotic to mitigate disease and improve dairy product development.

**TABLE 1:** Genomic features of the *Enterococcus faecium* MBBL3

Features	*E. faecium* MBBL3
Genome size (bp)	2,971,195
Genome coverage (×)	95.61
GC content (%)	37.97
No. of contigs	106
*N_50_* value (bp)	72,781
Genome completeness (%)	98.02 (0.17% contamination)
Coding sequences (CDSs)	2,954
Protein coding genes	2,843
RNA genes	76 (9 rRNA, 63 tRNA, and 4 ncRNA)
CRISPR array	1
Number of ARGs	2 [*aac(6')-II*, *msr(C*)]
Number of plasmids	2
Genome accession	JAZIFO000000000
SRA accession	SRR27558663

## Data Availability

The whole genome sequence of *Enterococcus faecium* MBBL3 has been archived in the NCBI GenBank and the NCBI Sequence Read Archive (SRA) under BioProject accession PRJNA1065156. The version reported in this study is identified as version JAZIFO000000000.1.
